# Role of a Pyroptosis-Related lncRNA Signature in Risk Stratification and Immunotherapy of Ovarian Cancer

**DOI:** 10.3389/fmed.2021.793515

**Published:** 2022-01-12

**Authors:** Zeyu Zhang, Zhijie Xu, Yuanliang Yan

**Affiliations:** ^1^Department of General Surgery, Xiangya Hospital, Central South University, Changsha, China; ^2^Department of Pathology, Xiangya Hospital, Central South University, Changsha, China; ^3^National Clinical Research Center for Geriatric Disorders, Xiangya Hospital, Central South University, Changsha, China; ^4^Department of Pharmacy, Xiangya Hospital, Central South University, Changsha, China

**Keywords:** ovarian cancer, pyroptosis, lncRNA, prognostic signature, tumor immune microenvironment

## Abstract

**Background:** Pyroptosis is a newly recognized form of cell death. Emerging evidence has suggested the crucial role of long non-coding RNAs (lncRNAs) in the tumorigenesis and progression of ovarian cancer (OC). However, there is still poor understanding of pyroptosis-related lncRNAs in OC.

**Methods:** The TCGA database was accessed for gene expression and clinical data of 377 patients with OC. Two cohorts for training and validation were established by random allocation. Correlation analysis and Cox regression analysis were performed to identify pyroptosis-related lncRNAs and construct a risk model.

**Results:** Six pyroptosis-related lncRNAs were included in the final signature with unfavorable survival data. Subsequent ROC curves showed promising predictive value of patient prognosis. Further multivariate regression analyses confirmed the signature as an independent risk factor in the training (HR: 2.242, 95% CI: 1.598–3.145) and validation (HR: 1.884, 95% CI: 1.204–2.95) cohorts. A signature-based nomogram was also established with a C-index of.684 (95% CI: 0.662–0.705). Involvement of the identified signature in multiple immune-related pathways was revealed by functional analysis. Moreover, the signature was also associated with higher expression of three immune checkpoints (PD-1, B7-H3, and VSIR), suggesting the potential of the signature as an indicator for OC immunotherapies.

**Conclusion:** This study suggests that the identified pyroptosis-related lncRNA signature and signature-based nomogram may serve as methods for risk stratification of OC. The signature is also associated with the tumor immune microenvironment, potentially providing an indicator for patient selection of immunotherapy in OC.

## Background

Ovarian cancer (OC) is a lethal gynecologic malignancy among all gynecological tumors (1). Surgery is recognized as the first-line treatment for OC; however, 4 patients out of 5 are unable to receive surgery because of advanced disease. Although new therapies other than chemotherapy are developed or under-developing for advanced OC, the 5-year survival rate improves with a relatively slow speed (2). The recognition of high- and low-risk patient groups may help individualize treatments, thus improving patient prognosis. Moreover, although immunotherapies improve patient prognosis of several cancers (3), there is currently no significant breakthrough in the development of immunotherapy in treating OC during the decade. A promising indicator is also needed to screen patients with OC who may benefit from certain immunotherapies

Pyroptosis, an inflammatory form of cell death triggered by certain inflammasomes (4), has been found to be related to multiple human diseases such as OC (5). Many genes are identified to play an important role in the processes of pyroptosis, such as NLRP3, which consists of the NLRP3/caspase-1 signaling pathway (6, 7). In the meantime, long non-coding RNAs (lncRNAs), members of the non-coding RNA family, also participate in the development of a variety of cancers (8). It is confirmed by many researchers that lncRNAs may have a complex impact on the development of OC (9). However, the importance of lncRNAs, which are associated with pyroptosis-related genes, is poorly investigated in OC.

The aim of this study is to identify prognostic pyroptosis-related lncRNAs, therefore establishing a promising signature and a signature-based nomogram for risk stratification in OC. The association between the signature and immune microenvironment is also investigated, as well as its role as an immunotherapy indicator.

## Materials and Methods

### Data Acquisition

Transcriptome and clinical data of 377 patients with OC were retrieved from the OV project of the TCGA database (http://cancergenome.nih.gov/), while the data of 88 normal tissues were retrieved from GTEx. Patients without adequate clinical data were excluded from the analyses.

### Identification of Pyroptosis-Related lncRNAs

A total of 33 pyroptosis-related genes were obtained from reports of Ye (Supplementary Table 1) (10). Pearson correlation test was performed to calculate correlations between lncRNAs and pyroptosis-related genes. A pyroptosis-related lncRNA was identified with Pearson correlation coefficient >0.3 and *p* < 0.001.

### Construction and Validation of the Signature

The cohort was randomly divided into a training cohort and a validation cohort at a 2:1 ratio. The data from the training cohort were used for constructing the prognostic pyroptosis-related lncRNAs signature, while the other was for validation. Univariate Cox regression analysis was performed to identify prognostic pyroptosis-related lncRNAs. Subsequently, the least absolute shrinkage and selection operator (LASSO) Cox regression was used to construct the signature, presented as follows: risk score = expression of lncRNA1 × β1lncRNA1 + expression of lncRNA2 × β2lncRNA2 + …expression of lncRNAn × βnlncRNAn. The two cohorts were further divided into the low-risk and high-risk groups, respectively. Survival analyses and time-dependent ROC curves were performed to investigate the prognostic value. Moreover, multivariate Cox regression of available patient characteristics was performed to reconfirm the prognostic value of the signature. In addition, a nomogram was constructed to predict patient prognosis more precisely.

### The mRNA-lncRNA Co-Expression Network

To better demonstrate the associations between pyroptosis-related genes and pyroptosis-related lncRNAs, a co-expression network was constructed. A Sankey diagram was used to illustrate mRNA-lncRNA relationships.

### Gene Set Enrichment Analysis and Subsequent Functional Enrichment Analyses

Tumor tissues were divided into the low-risk and high-risk groups based on risk scores. Differentially expressed genes between the groups were identified by the “DEseq2” package with cut-off criteria of false discovery rate <0.05 and |log_2_foldchange| > 1. After that, the differentially expressed genes were uploaded for GSEA analysis (http://www.broadinstitute.org/gsea) (11). The protein-protein interaction (PPI) chart was realized with the STRING database (12).

### Immunological Analysis

The abundance of tumor-infiltrating immune cells in OC tissues was investigated using the MCPcounter (13) and ssGSEA algorithms (14).

### Cell Culture

The human ovarian epithelial cell IOSE80 and ovarian cancer cells A2780 and TOV112D were obtained from Center for Molecular Medicine, Xiangya Hospital, Central South University. All cell lines were cultured in Dulbecco's Modified Eagle's Medium (DMEM; Gibco, Grand Island, NY, USA) with 10% fetal bovine serum (FBS; Gibco, Grand Island, NY, USA) and 1% penicillin/streptomycin (Gibco, Grand Island, NY, USA). The cultures were placed in a sterile incubator maintained at 37 C with 5% CO_2_.

### Quantitative PCR (qPCR)

Total RNA was extracted from disparate cell lines using a TRIzol reagent (Invitrogen, Carlsbad, CA, USA) following the protocol of the manufacturer and then converted to cDNA using a PrimeScriptTMRT reagent kit (6210; Takara, Dalian, China). A qPCR assay was performed with iTaqTM Universal SYBR Green Supermix (1725121; Bio-Rad, Hercules, CA, USA) to determine relative RNA levels. β-actin was used as an internal control for quantification of each gene. The sequences of gene primers are displayed in Supplementary Table 2. Relative expression levels of RNAs were determined using the 2^−ΔΔ*CT*^ method.

### Statistical Analyses

Statistical analyses were performed with the R 3.3.0 platform. One-way analysis of variance (ANOVA) and Welch's ANOVA were performed as appropriate. Generally, *p* < 0.05 was considered statistically significant.

## Results

### The Training and Validation Cohorts

**Figure 1:**
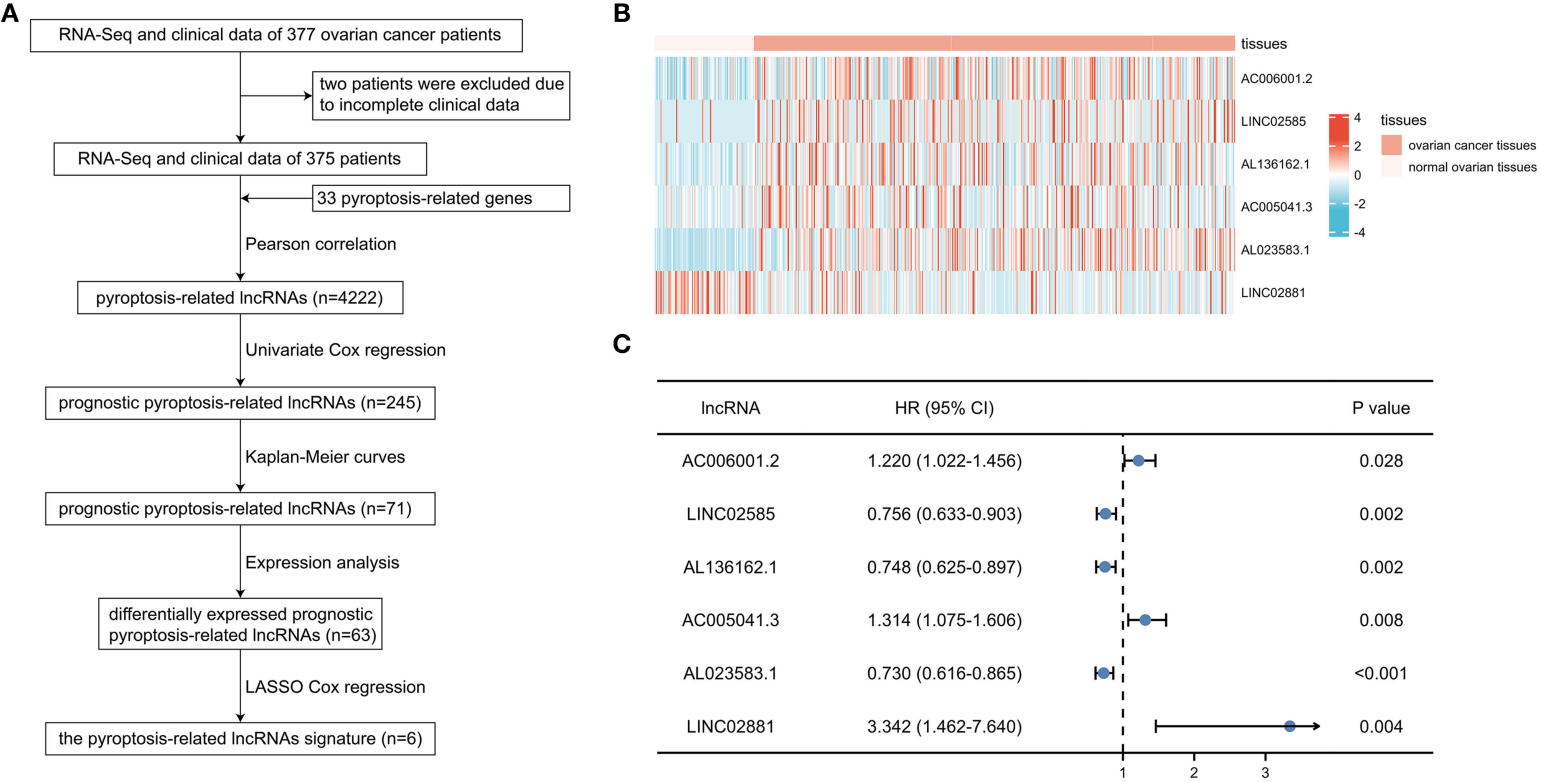
Identification of prognostic pyroptosis-associated long noncoding ribonucleic acids (lncRNAs) in patients with ovarian cancer (OC). **(A)** Flow chart of constructing the pyroptosis-related lncRNAs signature. **(B)** Heatmap of 6 prognostic pyroptosis-related lncRNAs in OC tissues and normal ovarian tissues. **(C)** Univariate Cox regression of 6 prognostic pyroptosis-related lncRNAs.

After excluding 2 patients without adequate clinical data (Figure 1A), the training cohort with 250 patients and the validation cohort with 125 patients were established by random allocation. Table 1 shows the patient and tumor characteristics of the cohorts. Except for neoplasm histologic grade, no difference was shown to have statistical significance.

**Table 1:** Characteristics of ovarian cancer patients in the training and validation cohorts.

**Characteristic**	**Training cohort** **(*n* = 250)**	**Validation cohort** **(*n* = 125)**	***P*-Value**
Age, median (IQR)	58 (50, 67)	61 (53, 71)	0.064
**Race**, ***n*** **(%)**			0.963
Asian	8 (3.3%)	3 (2.5%)	
Black or African American	16 (6.6%)	9 (7.4%)	
White	217 (89.3%)	109 (89.3%)	
Other	2 (0.8%)	1 (0.8%)	
**Anatomic subdivision**, ***n*** **(%)**			0.417
Unilateral	63 (26.9%)	38 (31.7%)	
Bilateral	171 (73.1%)	82 (68.3%)	
**Lymphatic invasion**, ***n*** **(%)**			0.689
No	34 (34%)	14 (29.2%)	
Yes	66 (66%)	34 (70.8%)	
**Neoplasm histologic grade**, ***n*** **(%)**			0.007
G1	0 (0%)	1 (0.8%)	
G2	35 (14.4%)	7 (5.7%)	
G3	208 (85.6%)	113 (92.6%)	
G4	0 (0%)	1 (0.8%)	
**Clinical stage**, ***n*** **(%)**			0.831
Stage I	1 (0.4%)	0 (0%)	
Stage II	16 (6.5%)	6 (4.8%)	
Stage III	195 (78.6%)	97 (78.2%)	
Stage IV	36 (14.5%)	21 (16.9%)	
**Chemotherapy**, ***n*** **(%)**			1.000
No	19 (7.6%)	10 (8%)	
Yes	231 (92.4%)	115 (92%)	
**Hormone therapy**, ***n*** **(%)**			0.408
No	230 (92%)	111 (88.8%)	
Yes	20 (8%)	14 (11.2%)	
**Targeted molecular therapy**, ***n*** **(%)**			0.847
No	227 (90.8%)	115 (92%)	
Yes	23 (9.2%)	10 (8%)	
**Immunotherapy**, ***n*** **(%)**			0.349
No	241 (96.4%)	123 (98.4%)	
Yes	9 (3.6%)	2 (1.6%)	

### Identification of Prognostic Pyroptosis-Related lncRNAs

A total of 4,222 pyroptosis-related lncRNAs were identified by Pearson correlation test, and subsequent univariate Cox regression and Kaplan-Meier curves were performed to determine the prognostic value of each lncRNA in the training cohort, suggesting 71 prognostic pyroptosis-related lncRNAs. After excluding 8 lncRNAs without significance in expression analyses, 63 candidate lncRNAs were included in the LASSO Cox regression analysis. Six pyroptosis-related lncRNAs (AC006001.2, LINC02585, AL136162.1, AC005041.3, AL023583.1, and LINC02881) were finally screened in the prognostic signature: risk score = (0.0624^*^AC006001.2 expression) + (−0.1014^*^LINC02585 expression) + (−0.1389^*^AL136162.1 expression) + (0.2398^*^AC005041.3 expression) + (−0.1734^*^AL023583.1 expression) + (2.1483^*^LINC02881 expression). The heatmap showed the expression level of the 6 lncRNAs in OC (Figure 1B), and the results of univariate regression analyses showed that the unfavorable prognostic value of AC006001.2, AC005041.3, LINC02881, and favorable prognostic value of LINC02585, AL136162.1, and AL023583.1 (Figure 1C).

**Figure 2:**
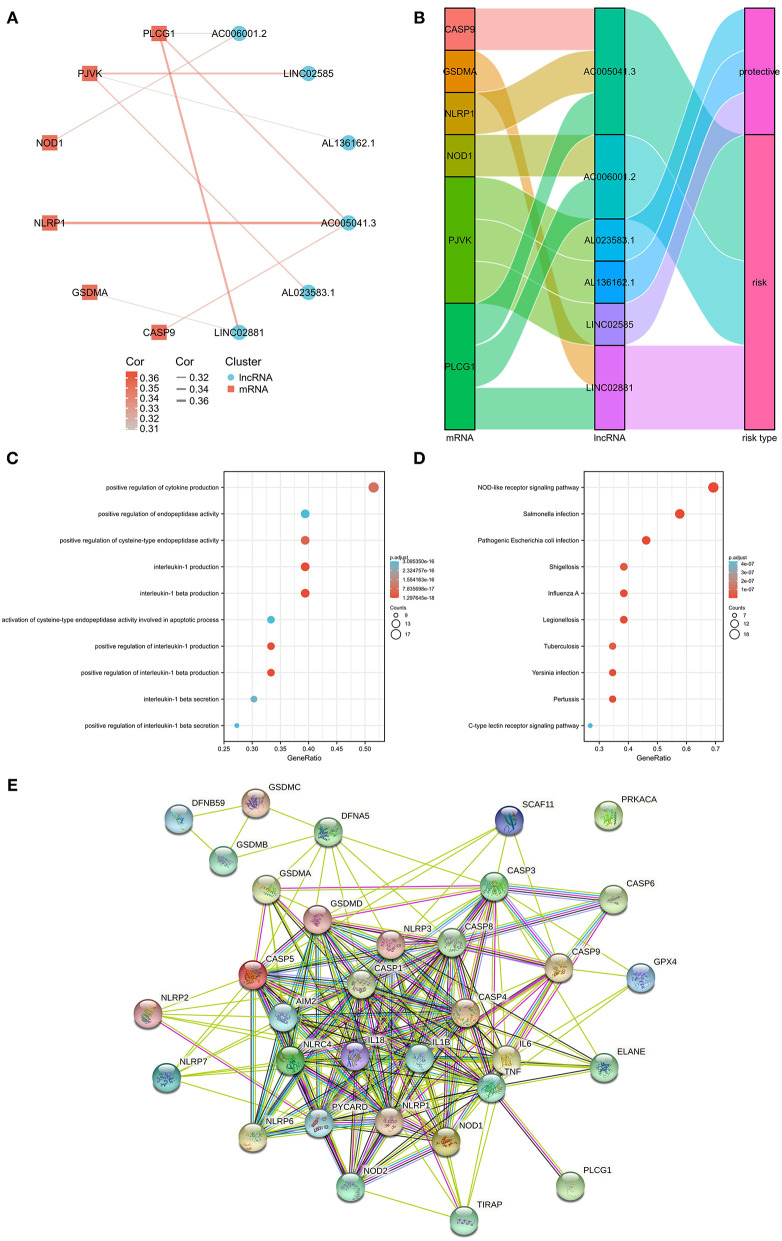
The messenger RNA (mRNA)-lncRNA co-expression network. **(A)** mRNA-lncRNA co-expression network of the pyroptosis-related genes and selected pyroptosis-related lncRNAs. **(B)** Sankey diagram showing the connection degree between the pyroptosis-related lncRNAs and the pyroptosis-related genes. **(C)** Gene Ontology (GO) analysis of 33 pyroptosis-related genes. **(D)** Kyoto Encyclopedia of Genes and Genomes (KEGG) analysis of 33 pyroptosis-related genes. **(E)** PPI chart of 33 pyroptosis-related genes.

Associations between the pyroptosis-related genes and lncRNAs were also investigated by co-expression analyses (Supplementary Table 3). Figure 2A shows the constructed network including 6 pyroptosis-related genes (CASP9, GSDMA, NLRP1, NOD1, PJVK, and PLCG1). It was noteworthy that AC005041.3 was associated with three pyroptosis-related genes, namely, CASP9, NLRP1, and PLCG1. Additionally, the Sankey diagram was also adopted to illustrate associations between genes and lncRNAs with their risk types (Figure 2B). Biological effects of 33 pyroptosis-related genes were also investigated, including GO (Figure 2C), KEGG (Figure 2D), and PPI chart (Figure 2E).

### Validation of Prognostic Pyroptosis-Related lncRNA Signature

**Figure 3:**
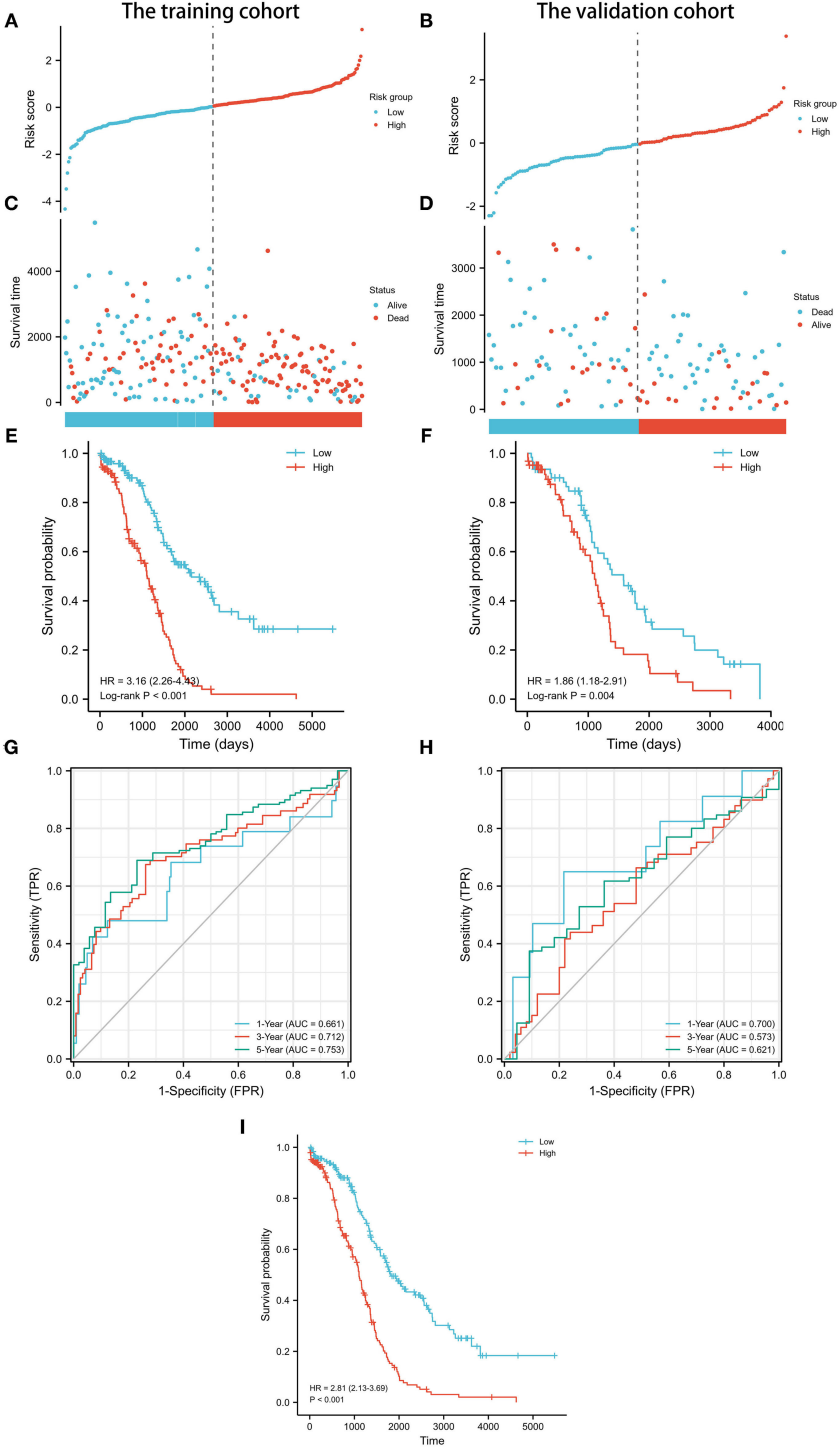
Prognostic analysis of pyroptosis-related lncRNA signature in the training and validation cohorts. **(A)** Distribution of risk scores in the training cohort. **(B)** Distribution of risk scores in the validation cohort. **(C)** Distributions of overall survival status, overall survival, and risk score in the training cohort. **(D)** Distributions of overall survival status, overall survival, and risk score in the validation cohort. **(E)** Kaplan-Meier curves for the overall survival of patients in the high- and low-risk groups in the training cohort. **(F)** Kaplan-Meier curves for the overall survival of patients in the high- and low-risk groups in the validation cohort. **(G)** AUC of time-dependent ROC curves verified the prognostic accuracy of the risk score in the training cohort. **(H)** AUC of time-dependent ROC curves verified the prognostic accuracy of the risk score in the validation cohort. **(I)** Kaplan-Meier curves for the overall survival of patients in the high- and low-risk groups in the whole cohort.

Next, the prognostic value of the signature was further examined in both the training and validation cohorts by Kaplan-Meier curves and time-dependent receiver operating characteristic (ROC) curves. After dividing the patients into the low-risk and high-risk groups (Table 2), worse survival data were shown among patients in the high-risk group in both cohorts (Figures 3A–D). Survival analyses further confirmed the unfavorable prognostic value of the signature in both cohorts (Figures 3E,F). In terms of ROC curves (Figures 3G,H), the area under curve (AUC) reached 0.681 at 1-year, 0.712 at 3-year, and 0.753 at 5-year in the training cohort, and 0.7 at 1-year, 0.573 at 3-year, and 0.621 at 5-year in the validation cohort. Survival analyses on the whole cohort also confirmed the unfavorable prognostic value of the signature (Figure 3I). Subsequently, the results of multivariate analyses confirmed the signature as an independent prognostic factor in both cohorts (Tables 3, 4). These results suggest a promising value of the signature in predicting prognosis of patients with OC. Additionally, the results of multivariate analysis of the risk score and the clinical characteristics in the whole cohort are shown in Table 5, while the clinical correlations between the clinical characteristics and risk score are shown in Table 6.

**Table 2:** Associations between risk score and characteristics of patients with ovarian cancer in the training cohort.

**Characteristic**	**Low-risk group** **(*n* = 125)**	**High-risk group** **(*n* = 125)**	***P*-Value**
Age, mean ± SD	57.84 ± 11.35	59.68 ± 11.52	0.205
**Race**, ***n*** **(%)**			0.188
Asian	4 (3.3%)	4 (3.3%)	
Black or African American	4 (3.3%)	12 (9.8%)	
Other	1 (0.8%)	1 (0.8%)	
White	112 (92.6%)	105 (86.1%)	
**Anatomic subdivision**, ***n*** **(%)**			0.812
Unilateral	31 (25.8%)	32 (28.1%)	
Bilateral	89 (74.2%)	82 (71.9%)	
**Lymphatic invasion**, ***n*** **(%)**			1.000
No	19 (34.5%)	15 (33.3%)	
Yes	36 (65.5%)	30 (66.7%)	
**Neoplasm histologic grade**, ***n*** **(%)**			0.815
G2	19 (15.3%)	16 (13.4%)	
G3	105 (84.7%)	103 (86.6%)	
**Clinical stage**, ***n*** **(%)**			0.114
Stage I	0 (0%)	1 (0.8%)	
Stage II	12 (9.6%)	4 (3.3%)	
Stage III	97 (77.6%)	98 (79.7%)	
Stage IV	16 (12.8%)	20 (16.3%)	
**Chemotherapy**, ***n*** **(%)**			0.056
No	5 (4%)	14 (11.2%)	
Yes	120 (96%)	111 (88.8%)	
**Hormone therapy**, ***n*** **(%)**			0.816
No	116 (92.8%)	114 (91.2%)	
Yes	9 (7.2%)	11 (8.8%)	
**Targeted molecular therapy**, ***n*** **(%)**			0.189
No	110 (88%)	117 (93.6%)	
Yes	15 (12%)	8 (6.4%)	
**Immunotherapy**, ***n*** **(%)**			0.500
No	119 (95.2%)	122 (97.6%)	
Yes	6 (4.8%)	3 (2.4%)	

**Table 3:** Univariate and multivariate analyses of risk factors and OS in the training cohort.

**Variables**	**HR (95% CI)**	***P*-Value**
**Univariate analyses**		
Age (years) (>60 vs. ≤ 60)	1.346 (0.973–1.863)	0.073
**Race**
White vs. Other	0.532 (0.216–1.306)	0.168
Asian vs. Other	0.779 (0.186–3.268)	0.733
Black or African American vs. Other	0.940 (0.320–2.759)	0.910
Anatomic subdivision (Bilateral vs. Unilateral)	1.243 (0.831–1.859)	0.290
Neoplasm histologic grade (G3–4 vs. G1–2)	1.092 (0.692–1.724)	0.705
Clinical stage (III and IV vs. I and II)	2.298 (0.849–6.221)	0.102
Chemotherapy (No vs. Yes)	2.351 (1.367–4.044)	0.002
Hormone therapy (Yes vs. No)	1.065 (0.633–1.792)	0.812
Targeted molecular therapy (Yes vs. No)	0.729 (0.394–1.348)	0.314
Immunotherapy (Yes vs. No)	0.812 (0.332–1.988)	0.649
Risk score (high-risk vs. low-risk)	2.314 (1.652–3.243)	<0.001
**Multivariate analyses**		
Chemotherapy (No vs. Yes)	2.058 (1.195–3.542)	0.009
Risk score (high-risk vs. low-risk)	2.242 (1.598–3.145)	<0.001

*HR, hazard ratio; CI, confidence interval; OS, overall survival; AJCC, American Joint Committee on Cancer*.

**Table 4:** Univariate and multivariate analyses of risk factors and OS in the validation cohort.

**Variables**	**HR (95% CI)**	***P*-Value**
**Univariate analyses**		
Age (years) (>60 vs. ≤ 60)	1.393 (0.899–2.159)	0.138
**Race**		
Black or African American vs. White	1.401 (0.641–3.061)	0.398
Asian vs. White	1.190 (0.163–8.674)	0.864
Other vs. White	0.543 (0.075–3.928)	0.545
Anatomic subdivision (Bilateral vs. Unilateral)	0.760 (0.474–1.218)	0.254
Neoplasm histologic grade (G3–4 vs. G1–2)	1.567 (0.631–3.890)	0.333
Clinical stage (III and IV vs. I and II)	1.628 (0.399–6.640)	0.497
Chemotherapy (No vs. Yes)	4.806 (2.241–10.307)	<0.001
Hormone therapy (Yes vs. No)	0.768 (0.405–1.454)	0.417
Targeted molecular therapy (Yes vs. No)	0.524 (0.227–1.213)	0.131
Immunotherapy (Yes vs. No)	0.400 (0.055–2.890)	0.364
Risk score (high-risk vs. low-risk)	1.931 (1.235–3.018)	0.004
**Multivariate analyses**
Chemotherapy (No vs. Yes)	4.552 (2.124–9.756)	<0.001
Risk score (high-risk vs. low-risk)	1.884 (1.204–2.950)	0.006

*HR, hazard ratio; CI, confidence interval; OS, overall survival; AJCC, American Joint Committee on Cancer*.

**Table 5:** Multivariate analyses of risk factors and clinical characteristics on OS.

**Variables**	**HR (95% CI)**	***P*-value**
Age (years)	1.022 (1.008–1.036)	0.002
Race (Not while vs. White)	1.833 (1.159–2.898)	0.010
Anatomic subdivision (Bilateral vs. Unilateral)	1.101 (0.798–1.520)	0.557
Neoplasm histologic grade (G3–4 vs. G1–2)	1.143 (0.737–1.774)	0.551
**Clinical stage**		
III vs. I and II	1.712 (0.742–3.947)	0.207
IV vs. I and II	2.025 (0.830–4.945)	0.121
Chemotherapy (No vs. Yes)	2.641 (1.635–4.265)	<0.001
Hormone therapy (Yes vs. No)	0.978 (0.607–1.577)	0.927
Targeted molecular therapy (Yes vs. No)	1.051 (0.627–1.760)	0.851
Immunotherapy (Yes vs. No)	0.842 (0.307–2.312)	0.739
Risk score (high-risk vs. low-risk)	1.774 (1.451–2.168)	<0.001

*HR, hazard ratio; CI, confidence interval; OS, overall survival; AJCC, American Joint Committee on Cancer*.

**Table 6:** Associations between risk score and characteristics of patients with ovarian cancer.

**Characteristic**	**Low-Risk group** **(*n* = 188)**	**High-Risk group** **(*n* = 187)**	***P*-Value**
Age, mean ± SD	58.25 ± 11.24	60.83 ± 11.41	0.028
**Race**, ***n*** **(%)**			0.538
Asian	6 (3.3%)	5 (2.7%)	
Black or African American	9 (4.9%)	16 (8.7%)	
Other	2 (1.1%)	1 (0.5%)	
White	165 (90.7%)	161 (88%)	
**Anatomic subdivision**, ***n*** **(%)**			0.297
Unilateral	47 (25.8%)	54 (31.4%)	
Bilateral	135 (74.2%)	118 (68.6%)	
**Lymphatic invasion**, ***n*** **(%)**			0.757
No	27 (34.2%)	21 (30.4%)	
Yes	52 (65.8%)	48 (69.6%)	
**Neoplasm histologic grade**, ***n*** **(%)**			0.775
G1	0 (0%)	1 (0.6%)	
G2	22 (11.9%)	20 (11.1%)	
G3	163 (88.1%)	158 (87.8%)	
G4	0 (0%)	1 (0.6%)	
**Clinical stage**, ***n*** **(%)**			0.104
Stage I	0 (0%)	1 (0.5%)	
Stage II	16 (8.5%)	6 (3.3%)	
Stage III	144 (76.6%)	148 (80.4%)	
Stage IV	28 (14.9%)	29 (15.8%)	
**Chemotherapy**, ***n*** **(%)**			0.020
No	8 (4.3%)	21 (11.2%)	
Yes	180 (95.7%)	166 (88.8%)	
**Hormone therapy**, ***n*** **(%)**			0.578
No	173 (92%)	168 (89.8%)	
Yes	15 (8%)	19 (10.2%)	
**Targeted molecular therapy**, ***n*** **(%)**			0.281
No	168 (89.4%)	174 (93%)	
Yes	20 (10.6%)	13 (7%)	
**Immunotherapy**, ***n*** **(%)**			0.546
No	181 (96.3%)	183 (97.9%)	
Yes	7 (3.7%)	4 (2.1%)	

### Signature-Based Nomogram

**Figure 4:**
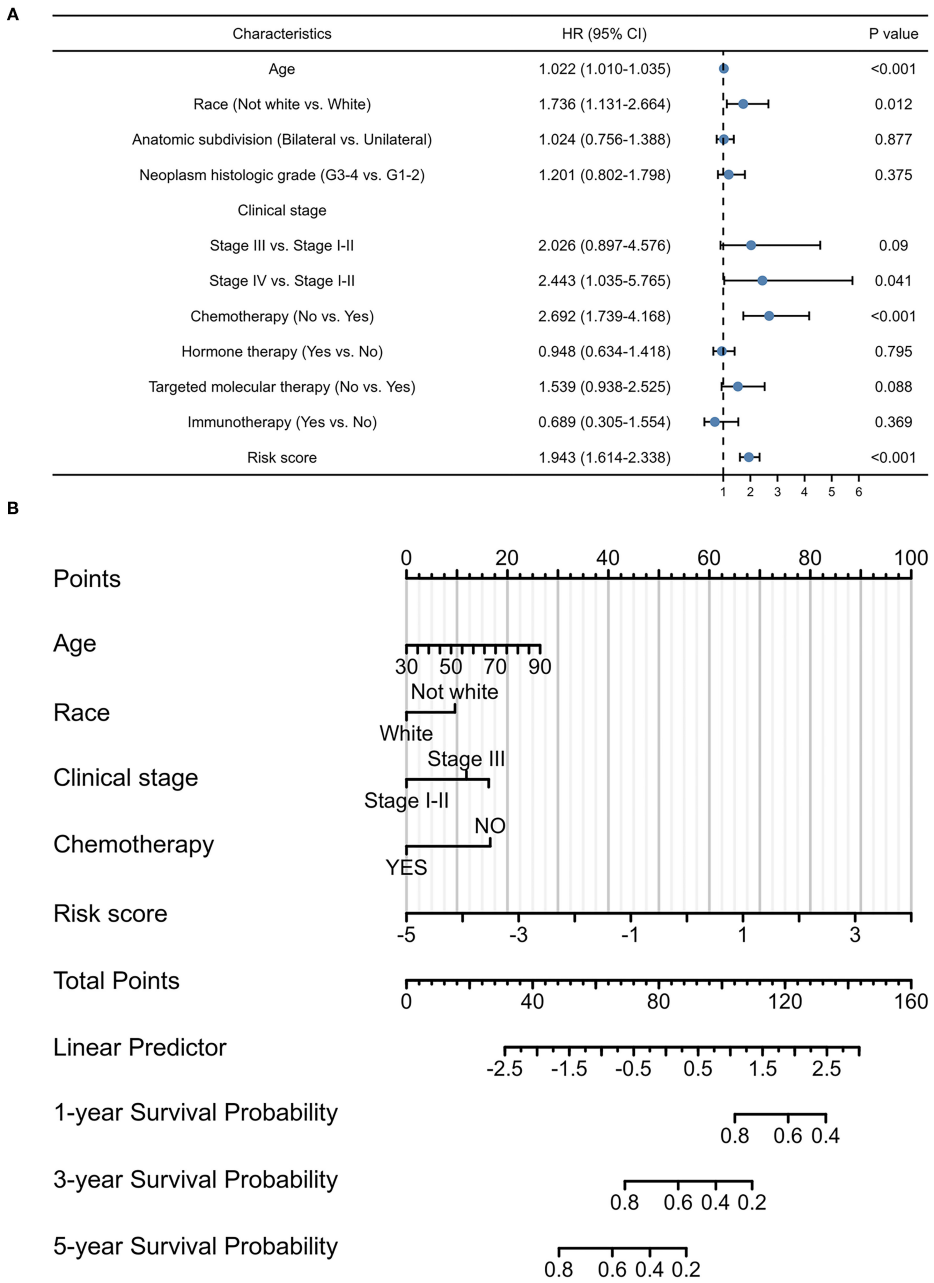
Prognostic values of the pyroptosis-related lncRNA signature. **(A)** Multivariate Cox regression of patient characteristics and the signature in the whole cohort. **(B)** Nomogram constructed using patient characteristics and the signature.

Considering the contributions of other characteristics in patient prognosis, an additional endeavor was made to construct a signature-based nomogram. After screening prognostic factors by univariate Cox regression analyses (Figure 4A), a nomogram, including pyroptosis-related lncRNA signature, age, race, clinical stage, and chemotherapy, was constructed to predict the 1-, 3-, and 5-year survival probability with a C-index of 0.684 (95% CI: 0.662–0.705) in OC (Figure 4B).

### Associations Between the Signature and Immune-Related Pathways

**Figure 5:**
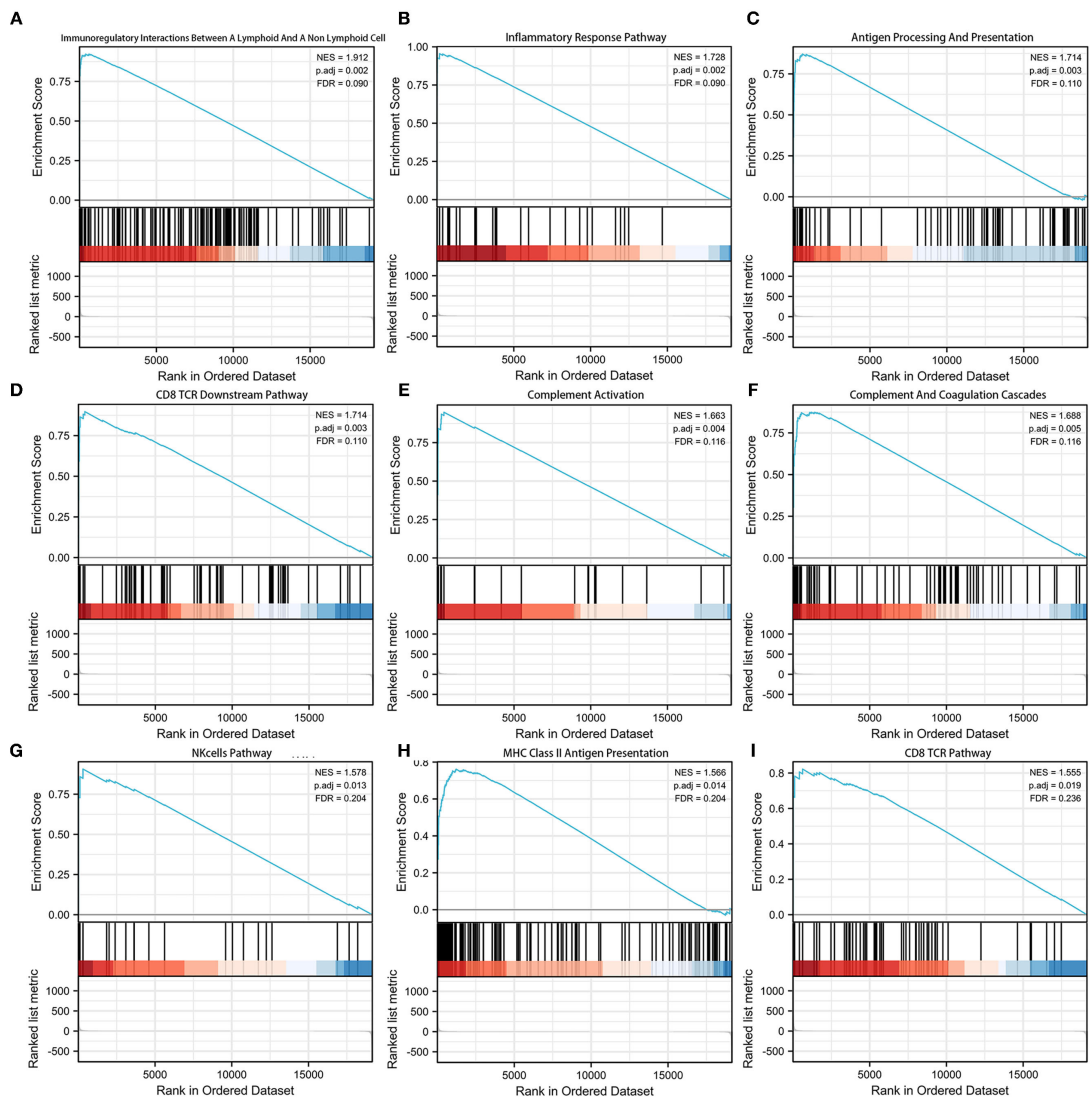
Gene set enrichment analysis (GSEA) of the pyroptosis-related lncRNA prognostic signature. **(A)** Immunoregulatory interactions between a lymphoid cell and a non-lymphoid cell. **(B)** Inflammatory response pathway. **(C)** Antigen processing and presentation. **(D)** CD8TCR downstream pathway. **(E)** Complement activation. **(F)** Complement and coagulation cascades. **(G)** NK cell pathway. **(H)** MHC class 1II antigen presentation. **(I)** CD8TCR pathway.

Gene set enrichment analysis (GSEA) was adopted to investigate the potential mechanism of the signature (Supplementary Table 4). Figure 5 shows the top 9 immune-associated signaling pathways, including immunoregulatory interactions between a lymphoid and a non-lymphoid cell, inflammatory response pathway, antigen processing and presentation, CD8 TCR downstream pathway, complement activation, complement and coagulation cascades, NK cell pathway, MHC class II antigen presentation, and CD8 TCR pathway. These findings suggest the potential associations between the identified signature and immune regulation.

### Associations Between the Signature and Immune Infiltration

**Figure 6:**
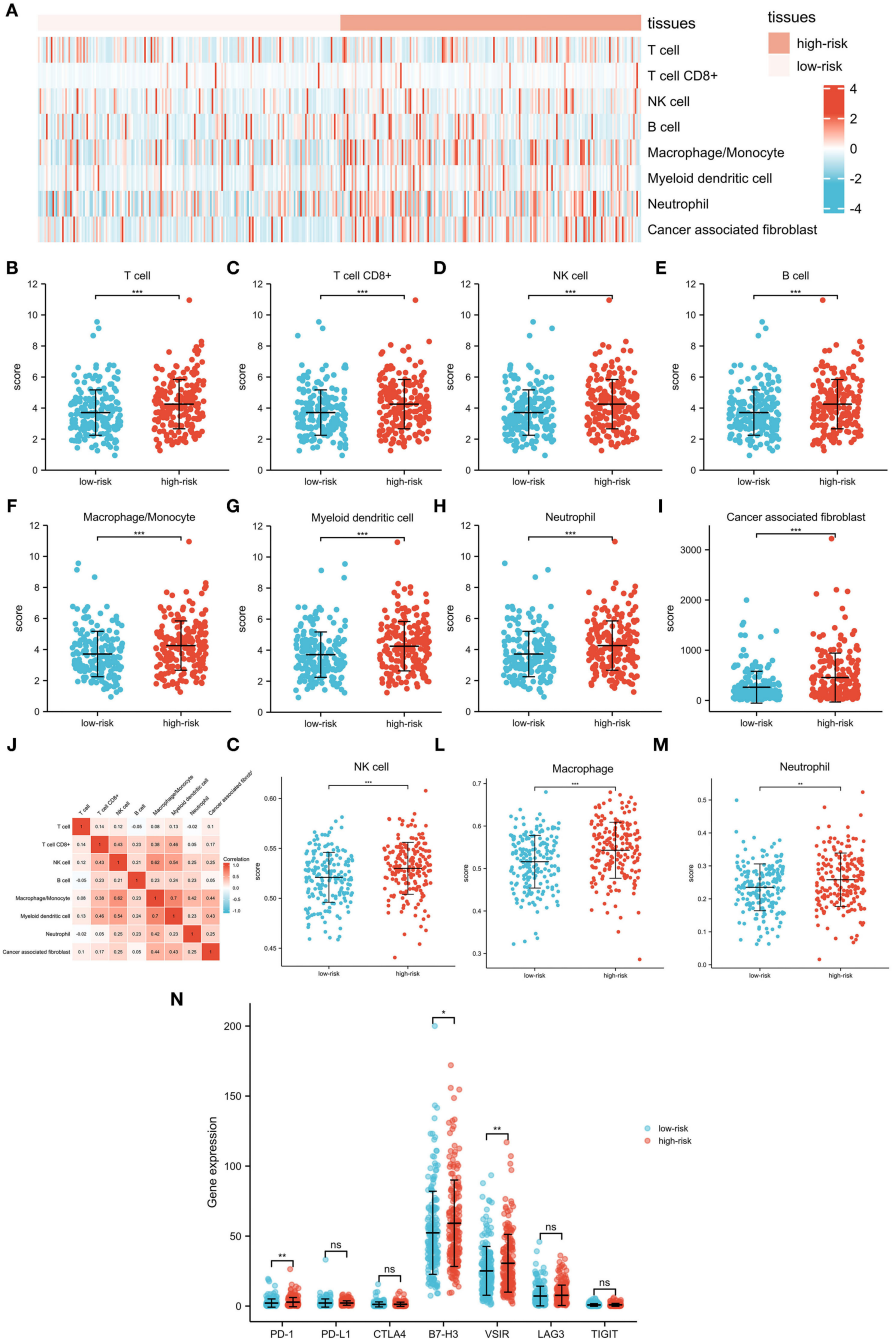
Interactions between the pyroptosis-related lncRNA signature and immune regulation in patients with OC. **(A)** Heatmap of the tumor-infiltrating cell in low-risk and high-risk patients. **(B)** Comparisons of T cell between the low-risk group and the high-risk group. **(C)** Comparisons of CD8 T cell between the low-risk group and the high-risk group. **(D)** Comparisons of NK cell between the low-risk group and the high-risk group. **(E)** Comparisons of B cell between the low-risk group and the high-risk group. **(F)** Comparisons of macrophage/monocyte between the low-risk group and the high-risk group. **(G)** Comparisons of myeloid dendritic cell between the low-risk group and the high-risk group. **(H)** Comparisons of neutrophil between the low-risk group and the high-risk group. **(I)** Comparisons of cancer-associated fibroblast between the low-risk group and the high-risk group. **(J)** Correlation matrix of immune cells in OC. **(K)** Comparisons of NK cell between the low-risk group and the high-risk group using the ssGSEA algorithm. **(L)** Comparisons of macrophage between the low-risk group and the high-risk group using the ssGSEA algorithm. **(M)** Comparisons of neutrophil between the low-risk group and the high-risk group using the ssGSEA algorithm. **(N)** Comparisons of multiple immune checkpoints between the low-risk group and the high-risk group, including PD-1, PD-L1, CTLA4, B7-H3, VSIR, LAG3, and TIGIT.

To further investigate associations between the signature and the immune microenvironment, the MCPcounter algorithm was used to calculate immune infiltration cells in OC. The difference in proportions of immune cells is illustrated in Figures 6A–I, and the relationships among different cells is illustrated in Figure 6J. T cells, CD8+ T cells, NK cell, B cell, macrophage/monocyte, myeloid dendritic cell, neutrophil, and cancer-associated fibroblast were shown to be upregulated in the high-risk group. Furthermore, we also applied the ssGSEA algorithm to recalculate immune infiltration cells in OC, which confirmed that proportions of NK cell, macrophage, and neutrophil were indeed upregulated in the high-risk group (Figures 6K–M). Moreover, comparisons of several common immune checkpoints expression levels showed that PD-1, B7-H3, and VSIR were expressed higher in the high-risk group than in the low-risk group (Figure 6N), suggesting the potential value of the signature as an immunotherapy indicator in OC.

### qRT-PCR

**Figure 7:**
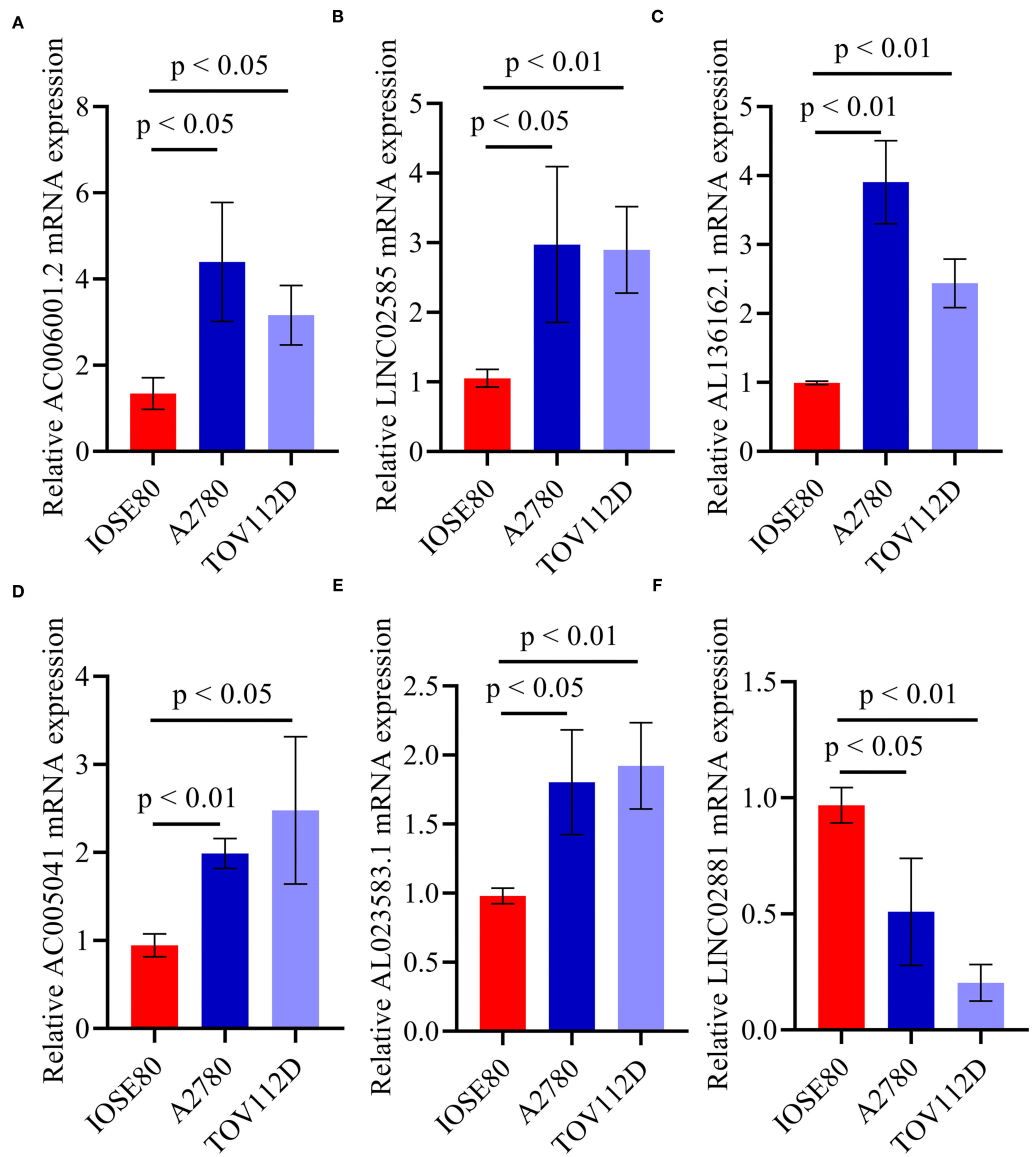
qRT-PCR of the six identified pyroptosis-related lncRNAs. **(A)** AC006001.2. **(B)** LINC02585. **(C)** AL136162.1. **(D)** AC005041.2. **(E)** AL023583.1. **(F)** LINC02881.

Quantitative real-time PRC (qRT-PCR) was performed to confirm the expression pattern of the six identified pyroptosis-related lncRNAs in two OC cell lines (Figure 7). The results confirmed that the expression levels of AC006001.2, LINC02585, AL136162.1, AC005041.3, and AL023583.1 were upregulated in OC, and that LINC02881 was downregulated, which was consistent with the results of the RNA-seq.

## Discussion

In this study, comprehensive analyses were performed to identify and investigate the prognostic pyroptosis-related lncRNAs in OC. A novel prognostic signature consisting of 6 pyroptosis-related lncRNAs (AC006001.2, LINC02585, AL136162.1, AC005041.3, AL023583.1, and LINC02881) and a signature-based nomogram were constructed and showed promising and validated results. Subsequent GSEA and immune microenvironment analyses all indicated the potential involvement of the signature in the immunology of OC. This study may provide new insights into risk stratification and immunotherapy development of patients with OC.

Long non-coding RNAs (lncRNAs) are recognized as key players in epigenetic regulation among patients with cancer (15), including patients with OC (16). However, few studies have focused on the role of lncRNAs in the pyroptosis of OC. Li et al. have previously reported that lncRNA GAS5 could suppress ovarian cancer by inducing inflammasome formation and pyroptosis (17). Moreover, the study by Tan et al. also indicated lncRNA HOTTIP targeted the miR-148a-3p/AKT2 axis, thus inhibiting ASK1/JNK signaling and NLRP1 inflammasome-mediated pyroptosis in ovarian cancer (18). In this study, 6 pyroptosis-related lncRNAs (AC006001.2, LINC02585, AL136162.1, AC005041.3, AL023583.1, and LINC02881) were identified to be associated with progression of OC, and none of them has been investigated previously. Future studies should be encouraged to reveal the underlying mechanisms of these candidate lncRNAs in OC biology, especially cell pyroptosis. Additionally, the 6 identified lncRNAs were found to be not associated with the other 27 pyroptosis-related genes. However, it should be pointed out that the false negative rate could also potentially cause the situation. Thus, future studies should also put efforts to validate the associations between the 6 identified lncRNAs and other pyroptosis-related genes.

Several immune checkpoint inhibitors (ICIs) have been applied to various cancers with promising results; however, unfortunately, there is currently no approved immunotherapy for OC. There are strong theoretical rationales to apply immunotherapy in OC, which presents an elevated number of tumor-infiltrating lymphocytes and neoantigen load (19). However, clinical results of two anti-PD-1 drugs (pembrolizumab and nivolumab) and three anti-PD-L1 drugs (atezolizumab, avelumab, and durvalumab) were all disappointing. Therefore, some strategies were developed aiming at sensitizing OC to immunotherapy by combining it with chemotherapy, anti-angiogenetics, poly ADP-ribose polymerase inhibitor, and radiotherapy (20). Patient selection can be another significant issue when applying immunotherapy in OC. Proper patient selection for each type of immunotherapy or proper immunotherapy selection for each patient is crucial for improving patient prognosis in cancers, including OC. In this study, three immune checkpoints (PD-1, B7-H3, and VSIR) were shown to be expressed higher among patients with high-risk score. B7-H3, also known as CD276, is an immune checkpoint molecule and immunoregulatory protein, which participates in tumor microenvironment shaping and development (21). VSIR, also known as VISTA, is a well-established immune regulatory receptor functioning like a homeostatic regulator that actively normalizes immune responses (22). On the one hand, since the immunotherapy of B7-H3 and VSIR has not been developed in OC, further studies developing immunotherapies in OC can focus on these two immune checkpoints. On the other hand, the identified signature in this study can serve as an indicator to select patients with OC who may benefit from immunotherapies of PD-1, B7-H3, and VSIR.

Pyroptosis is proven to be highly related to immune response, and recent evidence also reported the immunostimulatory function of pyroptosis, as well as a significant role in promoting the efficacy of cancer immunotherapy (23, 24). The roles of some lncRNAs in cancer immunotherapy are also well-elucidated by various studies (25). However, there has been no previous robust research reporting the role of the 6 identified lncRNAs (AC006001.2, LINC02585, AL136162.1, AC005041.3, AL023583.1, and LINC02881) in immune therapies. For the very first time, this study preliminarily identified the potential contributions of these lncRNAs in immune therapies. Future studies should validate the function of these lncRNAs in immunotherapies, and focus on the specific mechanisms of lncRNAs in influencing immunotherapies, especially among patients with OC.

However, this study has certain limitations. First, the cohorts in this study were mainly based on the TCGA database. Thus, practical data were needed for further validations of the pyroptosis-related lncRNA signature in patients with OC. Second, this study failed to investigate the underlying mechanisms for regulating the pyroptosis-related lncRNA signature in tumor immune response. Moreover, further studies should focus on the potential of the pyroptosis-related lncRNA signature as an indicator of immunotherapies.

## Conclusion

This study suggests that the identified pyroptosis-related lncRNA signature and the signature-based nomogram may serve as methods for risk stratification of OC. The signature is also associated with the tumor immune microenvironment, potentially providing an indicator for patient selection of immunotherapy in OC.

## Data Availability Statement

The original contributions presented in the study are included in the article/Supplementary Material, further inquiries can be directed to the corresponding author.

## Ethics Statement

Ethical review and approval was not required for the study on human participants in accordance with the local legislation and institutional requirements. Written informed consent for participation was not required for this study in accordance with the national legislation and the institutional requirements.

## Author Contributions

YY and ZX conceived the design of the study and edited the manuscript. ZZ and YY performed the study, collected the data, and contributed to the design of the study. ZZ, YY, and ZX prepared the manuscript. All the authors made substantive intellectual contributions to this study and read and approved the final version of the manuscript.

## Funding

This study was supported by grants from the National Natural Science Foundation of China (81803035), China Postdoctoral Science Foundation (2021T140754, 2020M672521), Natural Science Foundation of Hunan Province (2019JJ50932, 2020JJ5934), and Postdoctoral Science Foundation of Central South University (248485).

## Conflict of Interest

The authors declare that the research was conducted in the absence of any commercial or financial relationships that could be construed as a potential conflict of interest.

## Publisher's Note

All claims expressed in this article are solely those of the authors and do not necessarily represent those of their affiliated organizations, or those of the publisher, the editors and the reviewers. Any product that may be evaluated in this article, or claim that may be made by its manufacturer, is not guaranteed or endorsed by the publisher.

## Abbreviations

OC: ovarian cancer
lncRNAs: long non-coding RNAs
TPM: transcripts per kilobase million
LASSO: least absolute shrinkage and selection operator
GSEA: gene set enrichment analysis
ANOVA: one-way analysis of variance
AUC: area under curve.
