# Supplementary material for: Role of a Pyroptosis-Related lncRNA Signature in Risk Stratification and Immunotherapy of Ovarian Cancer
Source: Front Med (Lausanne). 2022 Jan 12;8:793515. doi: 10.3389/fmed.2021.793515 (PMC8791230; doi:10.3389/fmed.2021.793515)
Supplement: Supplementary file 2 [file Table_2.DOCX]

**Supplement Table S2**. The sequence of primers involved in this study.

| Gene | Sequence (5’-3’) | |
| --- | --- | --- |
| beta-actin | Forward | CATGTACGTTGCTATCCAGGC |
|  | Reverse | CTCCTTAATGTCACGCACGAT |
| AC006001.2 | Forward | ACCCTTCTGACACCGCTAAC |
|  | Reverse | CGTGCTCTTGCTAGTTCCCT |
| LINC02585 | Forward | CCCTCTCACCAATAGGGCTG |
|  | Reverse | ATTAGGAGCTGCCAATGGGG |
| AL136162.1 | Forward | ATCGTAGGACTTTGCTGCCC |
|  | Reverse | CCTCATGCTGGTGGCACATA |
| AC005041.2 | Forward | CACAGTTCTGGTCTCCGTCC |
|  | Reverse | GGATAGGGAGGGGGTCAGTT |
| AL023583.1 | Forward | TGCCTTTTGTTGCGCTTTTTCT |
|  | Reverse | GGTTGGGAAACGCTCATTGG |
| LINC02881 | Forward | TGCAGAGAAAGGTCCCGAG |
|  | Reverse | AGCCGTACAGAATCCACCTG |

RT, reverse transcription.
